# Initial assessment, level of care and outcome among children who were seen by emergency medical services: a prospective observational study

**DOI:** 10.1186/s13049-018-0560-8

**Published:** 2018-10-19

**Authors:** Carl Magnusson, Johan Herlitz, Thomas Karlsson, Christer Axelsson

**Affiliations:** 1 Department of Molecular and Clinical Medicine, Sahlgrenska Academy, University of Gothenburg, Gothenburg, Sweden; 2 Pre Hospen-Centre for Prehospital Research, Faculty of Caring Science, Work Life and Social Welfare, University of Borås, Borås, Sweden; 3 Health Metrics Unit, Sahlgrenska Academy, University of Gothenburg, Gothenburg, Sweden

**Keywords:** Triage, Children, Pre-hospital assessment, Patient safety, EMS nurse, Level of care

## Abstract

**Background:**

The assessment of children in the Emergency Medical Service (EMS) is infrequent representing 5.4% of the patients in an urban area in the western part of Sweden. In Sweden, patients are assessed on scene by an EMS nurse whom independently decides on interventions and level of care. To aid the EMS nurse in the assessment a triage instrument, Rapid Emergency Triage and Treatment System-paediatrics (RETTS-p) developed for Emergency Department (ED) purpose has been in use the last 5 years. The aim of this study was to evaluate the EMS nurse assessment, management, the utilisation of RETTS-p and patient outcome.

**Methods:**

A prospective, observational study was performed on 651 children aged < 16 years from January to December 2016. Statistical tests used in the study were Mann-Whitney U test, Fisher’s exact test and Spearman’s rank statistics.

**Results:**

The dispatch centre indexed life-threatening priority in 69% of the missions but, of all children, only 6.1% were given a life threatening RETTS-p red colour by the EMS nurse. A total of 69.7% of the children were transported to the ED and, of these, 31.7% were discharged without any interventions. Among the non-conveyed patients, 16 of 197 (8.1%) visited the ED within 72 h but only two were hospitalised. Full triage, including five out of five vital signs measurements and an emergency severity index, was conducted in 37.6% of all children. A triage colour was not present in 146 children (22.4%), of which the majority were non-conveyed. The overall 30-day mortality rate was 0.8% (*n* = 5) in children 0–15 years.

**Conclusions:**

Despite the incomplete use of all vital signs according to the RETTS-p, the EMS nurse assessment of children appears to be adapted to the clinical situation in most cases and the patients appear to be assessed to the appropriate level of care but indicating an over triage. It seems that the RETTS-p with full triage is used selectively in the pre-hospital assessment of children with a risk of death during the first 30 days of less than 1%.

## Background

The Emergency Medical Services (EMS) have developed rapidly over the last decades with the ability to perform interventions on scene which requires competence and clinical judgment to adequately assess the patient’s condition. In Sweden all ambulances are manned by a registered nurse, often with specialist training. The EMS nurses have been given the responsibility to assess patients at the scene and independently decide on treatment and level of care. To aid the EMS nurse in the assessment a triage protocol is being used. The Rapid Emergency Triage and Treatment System-paediatrics (RETTS-p) was initially developed for triaging within the paediatric Emergency Department (pED), and the majority of the pEDs at university hospitals use the RETTS-p. The pED in the western part of Sweden have approximately 50,000 visits each year with a hospital admission rate of 14% and have been using the RETTS-p since 2010. The EMS, organised under the university hospital, implemented the RETTS-p during 2014 to start the triage process at an early stage in the chain of care. The RETTS-p is a five-level scale including vital signs (VS) in each patient assessment. Five-level triage scales have shown a higher accuracy identifying critical ill patients compared to three-level triage scales [1, 2]. The Canadian paediatric triage and acuity scale (pedCTAS), Manchester triage system (MTS), Emergency severity index (ESI), and the Australasian triage scale (ATS) are all based on expert opinion such as the RETTS-p and are common in the pED [3]. They have shown moderate to good reliability and validity [4–11]. The ESI have in several studies in different locations shown an association of 80–100% predicting hospital admission in the highest triage levels [9, 12–14]. Validity studies are lacking for the RETTS-p but previous studies have shown a good to a very good reliability between nurses in the pED [15, 16]. The accuracy of triage in the EMS has been extensively studied in the assessment of trauma patients, indicating difficulties in the assessment of the patient with both over-triage and under-triage [17–20]. A five-level triage tool in the EMS might be favourable for detecting a severely ill patient [21]. The use of triage in the EMS to assess paediatric patients was proposed 25 years ago [22]. However, implementing more complex triage systems for adult patients has shown only moderate agreement between the EMS assessment and ED nurses [23, 24]. Furthermore, the appropriateness of the EMS nurse assessment utilising an pED triage system is unknown. Thus, there is a knowledge gap whether it is feasible to apply it in the pre-hospital setting in order to triage the patient to the appropriate triage level in the pED. The aim of this study was therefor to evaluate the initial priority given by the dispatcher,the EMS nurse assessment, management, utilisation of the RETTS-p and finally patient outcome in an unselected population of children under the age of 16.

## Methods

### Design

The present study was a prospective observational study of paediatric emergency patients who were assessed by an EMS nurse.

### RETTS-p

The RETTS-p is made up of two parts, vital signs (VS) and emergency signs and symptoms (ESS). The level of severity is based on the highest colour of ESS or VS that becomes the final triage level. Red is stated as life threatening, orange is potentially life threatening and both levels are, from a time perspective, defined as emergency care directly. Yellow and green are defined as no individual medical risk if put on wait for assessment by a physician. Five VS, respiratory rate, oxygen saturation, pulse frequency, body temperature and level of consciousness, are recorded and adjusted to age intervals, including pulse correction for patients presenting with fever. An upset child can affect the pulse rate, and this may prevent the child from showing a reliable VS [25]. The level of consciousness is defined by the RETTS-p as “alert” (Green), “tired/crummy” (Yellow), GCS 11–13 (Orange) and GCS ≤10 or ongoing seizures (Red). There are a total of 40 ESS cards with the most common complaints. The RETTS-p is not constructed as an instrument to decide whether the patient qualifies for emergency care nor to reject a patient emergency care in a pre-hospital context. However, the lowest triage level, blue category, means that, after assessment, the patient could be referred to other levels of care with no medical risk. Within the EMS organisation included in this study, only levels green to red are currently being used.

### Settings

**Fig. 1:**
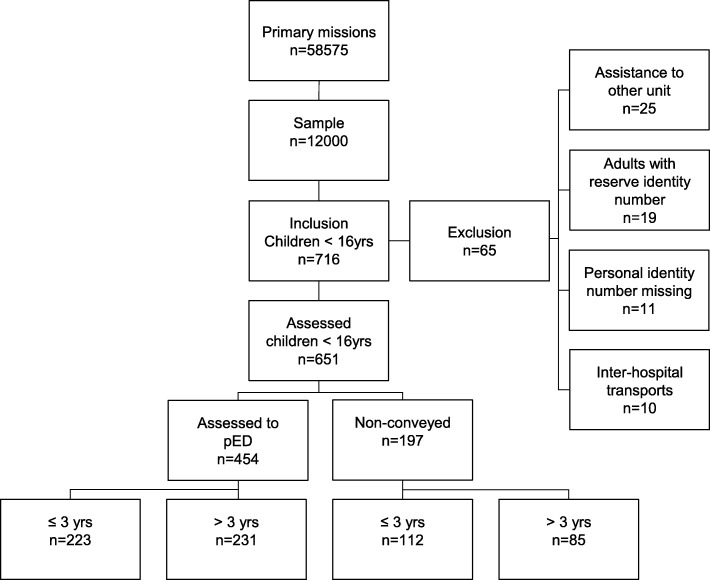
Flow chart of the studied patients, the distribution of patient assesment and median age

The study was conducted within an EMS organisation operating in an urban area in the western part of Sweden. The EMS organisation covers an area of 900 km^2^ with a population of 660,000 inhabitants and with predominantly short transportation times. During the year of 2016, the EMS carried out more than 80,000 ambulance missions (priority 1 to 3) and, of these, 58,575 assignments involve an initial patient assessment defined as a primary mission (Fig. 1). Approximately 3150 (5.4%) of these missions involve children aged < 16 years. The Emergency medical dispatch centre prioritise the patient with help from a Dispatch Medical Index (DMI). The DMI originally developed in the US has been adapted and used in Sweden since 1995 [26]. Based on the DMI a priority is given to the mission and an ambulance is allocated. Priority 1 is defined as life threatening to which the EMS responds with blue lights and sirens, priority 2 is urgent but not life threatening and priority 3 is assignments where the waiting time would not affect the medical condition. All the ambulances within the organisation are advanced life support (ALS) units and each ambulance is manned by at least one registered nurse responsible for patient assessment and the administration of drugs according to Swedish regulations. The EMS organisation aims to increase the number of specialist nurses trained in pre-hospital care, which comprises of a one-year postgraduate education in pre-hospital care. Fifty per cent of the staff within the EMS organisation are specialist nurses with this training.

### Materials

The patients in this study were included consecutively from a larger sample of the first 1000 assignments each month (January–December) in 2016 (*n* = 12,000). The inclusion criterion was an EMS assignment where a patient assessment took place at the scene. The exclusion criteria were as follows: 1) patients > 16 years, 2) assignments with no patient contact, 3) assignments assisting other EMS units and 4) inter-hospital transportation. A total of 716 patients under the age of 16 were initially identified from the sample. After a manual review, 65 patients were excluded as they did not fulfil the criteria and a total of 651 patients were finally included in the study (Fig. 1). Data were collected from EMS records and hospital records. Scanned patient records in which medications are prescribed in the pED were also reviewed for each patient.

### Statistical analysis

In the tables, results are presented as the number (percentage) or median (25th, 75th percentile). For two-group comparisons, the Mann-Whitney U test was used, while Fisher’s exact test was used for continuous/ordered and dichotomous/categorical variables respectively. To test for associations with age, Spearman’s rank statistics were used for continuous/ordered variables and the Mann-Whitney U test was used for dichotomous/categorical variables. The actual age (in years) was used for *p*-value calculations (Tables 1, 2, 3), although in Table 2 data are presented for patients dichotomised by the median (3 years). All tests are two sided and, due to the number of tests performed, *p*-values below 0.01 were regarded as statistically significant. Data processing and statistical analysis were performed using SPSS version 22.

**Table 1:** Children allocated to pED or non-conveyed

	Assessed to pED *n* = 454	Non-conveyed *n* = 197	*P*
Age - year (25th, 75th percentile)			
Median	4 (1,11)	2 (1,7)	0.002
Gender - n(%)			
Female	194 (42.7)	86 (43.7)	0.863
Dispatcher priority - n(%)			<0.001
Priority 1	336 (74.0)	113 (57.3)	
Priority 2	113 (24.9)	75 (38.1)	
Priority 3	5 (1.1)	9 (4.6)	
Dispatch classification index^a^ - n(%)			
Respiratory distress	89 (19.6)	51 (25.9)	0.078
Fever	55 (12.1)	34 (17.3)	0.083
Convulsions ongoing/ terminated	63 (13.9)	11 (5.6)	0.002
Major trauma high energy/ large bleeding/ headinjury	54 (11.9)	14 (7.1)	0.071
Minor trauma extremity/ wound/ fracture	46 (10.1)	21 (10.7)	0.888
Time of day - n(%)			0.034
08:00-16:00	163 (35.9)	58 (29.5)	
16:00-24:00	221 (48.7)	96 (48.7)	
24:00-08:00	70 (15.4)	43 (21.8)	
Medical history^b^ - n(%)			
No medical history	297 (65.4)	132 (67.0)	0.720
Asthma, common cold with asthma	27 (5.9)	15 (7.6)	0.487
Congenital disability	30 (6.6)	12 (6.1)	0.864
Febrile non-epileptical convulsions, absences	24 (5.3)	7 (3.6)	0.425
Allergies	18 (4.0)	8 (4.1)	0.999
Vital signs - median (25th, 75th percentile)			
Respiratory rate/min (141, 98)^c^	22 (18,30)	22 (20,29)	0.800
Saturation % (71, 72)	98 (97,100)	99 (98,100)	0.003
Pulse rate/min (79, 74)	117 (90,140)	115 (95,140)	0.858
Temperature °C (133, 86)	37.3 (36.7,38.4)	37.2 (36.7,38.1)	0.880
Level of consciousness according to RETTS-p - n(%) (81, 26)			<0.001
Alert	276 (74.0)	162 (94.7)	
Tired/ Crummy	54 (14.5)	8 (4.7)	
GCS 11-13	30 (8.0)	1 (0.6)	
GCS ≤ 10/ Ongoing seizure	13 (3.5)	0	
EMS nurse triage level according to RETTS-p - n(%) (64, 82)^c^			<0.001
Red	40 (10.3)	0 (0.0)	
Orange	109 (27.9)	4 (3.4)	
Yellow	179 (45.9)	34 (29.6)	
Green	62 (15.9)	77 (67.0)	
Assessed condition according to RETTS-p^d^ - n(%) (64, 83)^c^			
Trauma (head, thorax, extremity, burn)	100 (25.6)	17 (14.9)	<0.001
Respiratory difficulty (dyspnoea, Shortness of breath)	58 (14.9)	28 (24.6)	0.023
Convulsion	76 (19.5)	5 (4.4)	< 0.001
Fever	26 (6.7)	20 (17.5)	0.001
Abdominal pain	30 (7.7)	10 (8.8)	0.696
Drug administration - n(%)^e^			
EMS nurse drug administration	204 (44.9)	26 (13.2)	< 0.001
Antipyretics	74 (16.3)	11 (5.6)	< 0.001
Local anesthetics	43 (9.5)	0	< 0.001
Oxygen	29 (6.4)	1 (0.5)	< 0.001
Analgetics	29 (6.4)	0	< 0.001
Steroids	25 (5.5)	8 (4.1)	0.561
Mortality - n(%)			
< 30 days	5 (1.1)	0	0.330

^a^Dispatch index can consist of one or two indexes

^b^The most common medical history, a patient can have more than one condition

^c^Missing vital signs, triage level and assessed condition for children assessed to pED and non-conveyed respectively

^d^The most common assessed conditions according to RETTS-p

^e^The most common administered drugs, a patient could have been administered more than one

**Table 2:** Patient assessment and association with age

	Age ≤ 3 years *n* = 335	Age > 3 years *n* = 316	*P*
Gender - n(%)			
Female	133 (39.7)	147 (46.5)	0.030
Dispatcher priority - n(%)			<0.001
Priority 1	253 (75.5)	196 (62.0)	
Priority 2	75 (22.4)	113 (35.8)	
Priority 3	7 (2.1)	7 (2.2)	
Dispatch classification index^a^ - n(%)			
Respiratory distress	102 (30.4)	38 (12.0)	<0.001
Fever	64 (19.1)	25 (7.9)	<0.001
Convulsions ongoing/ terminated	49 (14.6)	25 (7.9)	0.146
Major trauma high energy/ large bleeding/ headinjury	32 (9.6)	36 (11.4)	0.243
Minor trauma exremity/ wound/ fracture	19 (5.7)	48 (15.2)	<0.001
Time of day - n(%)			0.015
08:00-16:00	103 (30.7)	118 (37.3)	
16:00-24:00	166 (49.6)	151 (47.8)	
24:00-08:00	66 (19.7)	47 (14.9)	
Medical history^b^ - n(%)			
No medical history	230 (68.7)	199 (63.0)	0.015
Common cold with asthma/ asthma	25 (7.5)	17 (5.4)	0.954
Congenital disability	15 (4.5)	27 (8.5)	0.159
Febrile non-epileptical convulsions, absences	24 (7.2)	7 (2.2)	0.168
Allergies	11 (3.3)	15 (4.7)	0.178
Primary vital signs - median (25th, 75th percentile)			
Respiratory rate/min (164, 75)^c^	30 (24,40)	20 (16,22)	<0.001
Saturation % (103, 40)	98 (97,100)	99 (98,100)	0.001
Pulse rate/min (113, 40)	140 (120,160)	98 (83,117)	<0.001
Temperature °C (126, 93)	37.5 (36.7,38.8)	37.1 (36.7,37.8)	<0.001
Level of consciousness according to RETTS-p - n(%) (58, 49)			<0.001
Alert	204 (73.6)	234 (87.7)	
Tired/ Crummy	47 (17.0)	15 (5.6)	
GCS 11-13	19 (6.9)	12 (4.5)	
GCS ≤ 10/ Ongoing seizure	7 (2.5)	6 (2.2)	
EMS nurse triage level according to RETTS-p - n(%) (99, 47)^c^			0.134
Red	19 (8.1)	21 (7.8)	
Orange	52 (22.0)	61 (22.7)	
Yellow	93 (39.4)	120 (44.6)	
Green	72 (30.5)	67 (24.9)	
Assessed condition according to RETTS-p^d^ - n(%) (100,47)^c^			
Trauma (head, thorax, extremity, burn)	35 (14.9)	82 (30.5)	<0.001
Respiratory difficulty (dyspnoea, shortness of breath)	64 (27.2)	22 (8.2)	<0.001
Convulsions	55 (23.4)	26 (9.7)	0.002
Fever	29 (12.4)	17 (6.3)	0.023
Abdominal pain	5 (2.1)	35 (13.0)	<0.001
EMS nurse Assessment - n(%)			
Assessed to pED	223 (66.6)	231 (73.1)	0.002
Renewed contact within 72h^e^	10 (8.9)	6 (7.1)	0.511
Drug administration - n(%)^f^			
EMS nurse administration	114 (34.0)	116 (36.7)	0.085
Antipyretics	42 (12.5)	43 (13.6)	0.198
Local anesthetics	18 (5.4)	25 (7.9)	0.092
Oxygen	18 (5.4)	12 (3.8)	0.292
Analgesics	3 (0.9)	26 (8.2)	<0.001
Steroids	16 (4.8)	17 (5.4)	0.441
Mortality - n(%)			
< 30 days	3 (0.9)	2 (0.6)	0.404

^a^Dispatch index can consist of one or two indexes

^b^The most common medical history, a patient can have more than one condition

^c^Missing vital signs, triage level and assessed condition for ≤ 3 year and over 3 years respectively

^d^The most common assessed conditions according to RETTS-p

^e^Patients initially non-conveyed that visited the pED within 72 hours

^f^The most common administered drugs, a patient could have been administered more than one

**Table 3:** Adherence to the RETTS-p

	Full triage *n* = 245	Non-full triage *n* = 406	*P*
Age - year (25th, 75th percentile)			
Median	6 (2,12)	2 (1,7)	< 0.001
Gender - n(%)			
Female	109 (44.5)	171 (42.1)	0.568
Dispatcher priority - n(%)			0.862
Priority 1	170 (69.4)	279 (68.7)	
Priority 2	70 (28.6)	118 (29.1)	
Priority 3	5 (2.0)	9 (2.2)	
EMS nurse assessment - n(%)			
Non-conveyed	55 (22.4)	142 (35.0)	0.001
Renewed contact within 72h^a^	5 (9.1)	11 (7.7)	0.795
EMS nurse triage level according to RETTS-p - n(%) (0, 146)^b^			0.607
Red	15 (6.1)	25 (6.2)	
Orange	56 (22.9)	57 (14.0)	
Yellow	116 (47.3)	97 (23.9)	
Green	58 (23.7)	81 (20.0)	
Drug administration - n(%)			
EMS nurse administration	101 (41.2)	129 (31.8)	0.015
pED administration^c^	34 (17.9)	39 (14.8)	0.372
EMS nurse and pED administration^d^	41 (21.6)	61 (23.1)	0.701
Management pED - n(%)			
No intervention ⇒ discharged	53 (27.9)	91 (34.5)	0.153
Intervention prescription/min. surgery/x-ray ⇒ discharged	85 (44.7)	102 (38.6)	0.209
Admission to in-patient care	52 (27.4)	71 (26.9)	0.915
Days of in-patient care - n			
Mean (SD)	3.3 (6.1)	3.4 (4.7)	0.095
Median (25th,75th percentile)	2 (1,3)	2 (1,4)	0.674
ICD-10 Codes - n(%)^e^ (69, 170)^b^			
(S,T) Injury, poisoning and certain other consequnces of external causes	41 (23.4)	85 (35.9)	0.007
Fractures	8 (19.5)	14 (16.5)	
Superficial injuries	2 (4.9)	16 (18.8)	
(R) Symptoms, signs and abnormal clinical and laboratory findings	53 (30.3)	54 (22.8)	0.090
Convulsions, not elsewhere classified	25 (47.2)	35 (64.8)	
Unspecific abdominal pain	10 (18.9)	3 (5.5)	
(J) Diseases of the respiratory system	23 (13.1)	33 (13.9)	0.885
Acute obstructive laryngitis	6 (26.1)	11 (33.3)	
Acute upper respiratory infections	5 (21.7)	10 (30.3)	
(A,B) Certain infectious and parasitic diseases	16 (9.1)	22 (9.3)	0.999
Viralinfection of unspecified site	7 (43.8)	15 (68.2)	
Infectious gastroenteritis and colitis, unspecified	4 (25.0)	6 (27.3)	
Mortality - n (%)			
< 30 days	1 (0.4)	4 (0.9)	0.655

^a^Patients initially non-conveyed

^b^Missing triage level and diagnose code for full triage and non-full triage

^c^Administration of drugs in the pED identical to drugs in the ambulance and not administrated in the ambulance

^d^Administration of drugs in the ambulance and follow up dose or additional drugs in the pED identical to the drugs in the ambulance

^e^The five most common diagnose groups of patients transported to the pED

## Results

### Children allocated to pED or non-conveyed

The median age for all children was 3 years. Among children < 16 years of age, 30.3% were non-conveyed after assessment, with a referral to primary care or with self-care advice. These non-conveyed children were significantly younger than those transported to the pED (Table 1). There was a significant association between a higher priority level assessed by dispatch and the patient being transported to the pED. Thus, children assessed as being in need of pED care by the EMS nurse were given a significantly higher priority by dispatch. However, of the assignments with priority 1 from dispatch, only 7.8% were assessed as life threatening (RETTS-p red) by the EMS nurse and, in the total study population, only 6.1% of the patients were given a red triage level (Table 1). Respiratory distress was the most common DMI overall. The DMI for convulsions was significantly more common in the pED care group compared with those non-conveyed. There was no significant difference in EMS allocation regarding time of day between the two groups, but an overall decrease in patient contact during the night was observed. The majority of the children had no previous medical history, but, among those with a medical history, the most frequent previous conditions were asthma/common cold with asthma and congenital disability. Children assessed to the pED had a significantly greater reduction in oxygen saturation and a more affected level of consciousness than children who were not conveyed. In overall terms, a triage colour was missing in 22.4% of the assessments and the proportion was higher for children assessed to stay at the scene compared with patients transported to the pED. Patients assessed to pED care were associated with a significantly higher triage level. Trauma was the most common condition according to the RETTS-P assessment and, together with convulsions, it was significantly more common in those transported to the pED, whereas fever was more common in the non-conveyance group. Significantly more children in the pED group received medical treatment by the EMS nurse. Children who died within 30 days (*n* = 5) were all initially transported to hospital (Table 1).

### Renewed contact within 72 h

Of the non-conveyed patients (*n* = 197), 16 (8.1%) visited the pED within 72 h. Seven of these patients were transported by ambulance, one in a single manned technician patient transport and the remaining eight by their own transport. Three of the 16 patients were assessed, treated and discharged by a pED nurse, one patient was left before evaluation and 12 children were assessed by a physician. Of these 12, two were admitted to inpatient care, seven were discharged with an intervention/prescription and three patients were discharged without any intervention. One patient with a final diagnosis of anaphylaxis who initially received epinephrine, administered by one of the parents, was observed in the pED and was given additional treatment.

### Patient assessment and association with age

There was a significant inverse association between age and dispatch priority. Respiratory distress and fever were the most common DMI among younger children and they were significantly associated with a lower age. For older children, minor trauma was the most common condition assessed by dispatch and it was significantly associated with a higher age (Table 2). There was no significant difference in age regarding time of day. Increased respiratory rate, decreased oxygen saturation and increased pulse frequency and body temperature were all significantly associated with a lower age, as was a lower level of consciousness (Table 2). The RETTS-P assessment of respiratory difficulty and convulsions was significantly associated with a lower age, whereas trauma and abdominal pain were associated with increasing age (Table 2). The most frequently administered drug was acetaminophen. The administration of analgesics (morphine, fentanyl and esketamine) was significantly associated with a higher age (Table 2).

### Adherence to the RETTS-p

Of all the children assessed by the EMS nurse, 406 (62.4%) did not receive a full-triage (5 VS + ESS) and, of these children, 146 did not receive any triage colour at all (Table 3). Children with a limited triage were significantly younger and were non-conveyed to a greater extent. The most common missing VS was respiratory rate (Table 1), which was also more frequently missing among children who stayed at the scene compared with transported children (49.7 and 31.1% respectively, *p* < 0.001). There was no significant difference between the full triage and limited triage groups regarding admission to inpatient care or interventions in the pED (Table 3). A total of 412 (90.7%) patients transported to the pED received a diagnosis according to the ICD-10. Among all these patients, the most frequent diagnosis groups were “injury, poisoning and certain other consequences of external causes” and these diagnosis groups were significantly more common among children who had a limited triage compared with those with a full triage (Table 3).

## Discussion

Assignments involving children in pre-hospital emergency care are infrequent when related to the total number of ambulance missions in a national (Sweden) and an international perspective [27–30]. The use of a structured triage system supporting the EMS nurse in the assessment of children may therefore be advocated to assure a systematic assessment. However, one third of the patients in the study stayed at the scene, indicating low resource utilisation. These data suggest that management by other levels of care may be a feasible alternative in many cases and this has also previously been reported [31–34]. It is also known that dispatch priorities diverge from EMS assessments at the scene, resulting in dispatch over-triage [26, 35]. This was also found in this study. However, there may be several reasons for the high dispatch priority. The operator at the dispatch centre has limited possibilities to assess the child over the phone, is restricted to the DMI and should decide on the priority within seconds according to dispatch regulations. The patient’s condition is also a dynamic process where for example convulsions may have terminated at the arrival of the EMS. The available ambulance units may also play a role, where units with lower priority assignments can be cancelled and assigned to priority 1. Hence, if assigned to a lower priority, the time frame upon arrival may be uncertain for an ambulance to arrive at the scene. Furthermore, the EMS nurse at the scene administered drugs in 35% of patients for example inhalation drugs for acute obstructive laryngitis and the downgrade in priority on scene may also be explained by the fact that the intervention on scene lowered the triage level.

The diversity in the assessed severity of the condition between dispatch centre and the EMS indicates that other units, such as a single-responder unit, manned by one specialist trained and experienced nurse, may be a suitable alternative for the initial assessment of some of these patients. However, previous studies also report that patients of all ages who were assessed to stay at the scene with or without triage protocols renew their contact with health care within 72 h in up to 19% of cases [36, 37]. In this study, only 8.1% renewed their contact within 72 h, with only two patients admitted to inpatient care. This indicates that, with few exceptions, the EMS nurse has the ability and knowledge to assess and allocate patients to other levels of care. Furthermore, another 31.7% of the patients who were transported to the pED were discharged by the pED nurse or by a physician without any intervention. Parental concern about their child’s acuity and the feeling of security when transported by the EMS has been reported, even though it is not warranted [38], and this may sometimes explain why the EMS and the pED are contacted. A primary care centre located near the pED could be an alternative for this patient group. Another aspect is that more children who were non-conveyed were younger and, according to the EMS nurse, they could be managed by either self-care advice or treatment at the scene. On the other hand, the patients who were older were more predisposed to trauma and were therefore assessed as requiring pED resources due to for example a suspicion of a fracture.

Younger children were more frequently excluded from a triage colour, which could illustrate the difficulty of triaging an infant with VS according to the RETTS-p and the fact that this group were more likely to be non-conveyed. Patients with for example acute obstructive laryngitis where the primary RETTS-p assessment would indicate red or orange colour, were often thoroughly examined, treated and not conveyed. However, if triaged according to RETTS-p and only transported to the pED may have led to increased resource allocation in the pED.

This may indicate over-triage and the EMS nurse competence level, interventions on scene and the dynamic process of patient symptoms corresponding to the triage level in the EMS and later in the pED may play a role. Previous studies of the MTS similarly constructed as the RETTS-p have shown relatively high over-triage in the pED of 40–59% [10, 11, 39, 40], where over-triage was mostly found in the lower triage categories [10]. There was a certain non-compliance to the RETTS protocol leading to missing variables. This may be a sign of mismatch between the protocol and the patient population. Thus the nurse overruled the protocol by clinical judgement, which sometimes may highlight the soundness of clinical management. This is why it may be wise to have a specialized nurse instead of a technician that may always follow an algorithm. The priorities of a triage system should be aimed at a high sensitivity to reduce under-triage for safety reasons. However, an excess of over-triage could have an impact on resource utilisation and pose a threat to safety for other patients. On the other hand, if the RETTS-p is already correctly applied in the pre-hospital setting, it is possible to downgrade the triage level for patients who are improved by an EMS nurse intervention. Furthermore, the mere definition of a triage tool is to determine who needs emergency care from a physician directly and who can wait, which does not include any decision on treatment and release. A computerised decision support tool may be introduced to guide the EMS nurse in the assessment, thus applying triage protocols when indicated for the utilisation of pED resources, which may increase compliance in the assessment process [41, 42].

The majority of the children were not completely triaged according to the RETTS-p. These patients did not receive a triage colour at all or were given a triage colour but with one or more VS missing. It could be suggested that an incomplete triage could jeopardise patient safety and thus omit significant abnormal vital signs. However, previous studies have shown that VS are only moderate predictors when assessing children in need of a trauma centre [43] and, furthermore, that there is a correlation between the degree of acuity level and the number of VS that were registered [44]. Even though measuring VS in the EMS care of injured and sick children has been emphasised to reduce under-triage [45, 46], implementing VS in all the patients in the MTS for paediatrics did not improve performance [47]. There are a number of reasons that may explain why not all VS according to the RETTS-p were measured in this study. They include a lower age, patients assessed as not being in need of VS measurement, short transport times and isolated and minor injuries in otherwise healthy children. This indicates that the RETTS-p is not feasible in the pre-hospital setting for all patients since it is not applied systematically. Other instruments such as the Patient assessment triangle (PAT) may be a viable alternative in the pre-hospital setting together with an A-E survey [48]. The PAT is a less complex tool and has shown to be reliable, accurate and easy to use at the initial assessment to evaluate the clinical status [49–51]. There is a concern that a relatively high proportion of the younger children in this study not had all their VS recorded. Serious infections such as sepsis and meningitis have a higher incidence with decreasing age [52]. A previous study reported that 15.4% of the children, presented at the pED with a suspected infection, were diagnosed with a serious infection which were significantly associated with deviation in VS [53]. Even though not all five VP were recorded according to RETTS-p in this study, it may be that when VS were reported by the EMS nurse it was clinically relevant in the current situation. Such a hypothesis is supported by the low frequency of renewed contact within 72 h and the fact that of those, only one patient was diagnosed with a potentially life-threatening diagnosis with no deviation in VS. Despite being implemented in the EMS as a triage instrument, the RETTS-p was never intended to assess whether or not patients could remain at the scene. The aim was to use it during transport to the pED and, among these patients, to estimate the time required until the patient needed to be examined by a physician based on the severity of the condition. This may reflect why so many patients who stayed at the scene lacked a triage colour. Furthermore, some patients did not receive a triage colour due to a severe critical condition (cardiac arrest, obstructed airway). In these cases, the activation of a medical/trauma team and the requirement of an examination by a physician immediately upon arrival was obvious. Five patients died within thirty days from EMS arrival in this study. Three patients were hospitalized and of those two patients died from a terminal disease.

### Strengths and limitations

The major strength is that data were collected from a relatively large study cohort from a well-defined area in a systematic fashion, where data were prospectively reported. Major limitations were that data were collected from a single site in an urban setting with short transportation times which most likely influenced the results. The extrapolation of these data to other areas may therefore be problematic. Furthermore, due to the design of the study, data had to be collected from the patient records and important clinical parameters such as VS may have been measured but never recorded.

## Conclusions

A representative study cohort of children below 16 years of age appeared to be safely assessed by the EMS nurse either to stay at the scene in one third of the cases or to be transported to the paediatric emergency department, regardless of whether the triage instrument was used to its full extent. However, one third of the patients were discharged from the pED with no interventions indicating an over-triage. The RETTS-p triage protocol may act as a tool to guide the EMS nurse in the assessment with application when feasible. The development of the RETTS-p triage protocol incorporated into a computerised decision support system, and the implementation of nurse-manned units specialising in children may further increase the potential to assess children and refer them to other levels of care, such as primary care. This would hopefully result in reduced overcrowding at the paediatric emergency department and more efficient resource utilisation with preserved patient care and safety.

## Abbreviations

ALS: Advanced life support
ATS: Australasian triage scale
DMI: Dispatch medical index
EMS: Emergency medical services
ESI: Emergency severity index
ESS: Emergency signs and symptoms
GCS: Glasgow coma scale
ICD-10: International statistical classification of diseases and related health problems – tenth revision
MTS: Manchester triage system
PAT: Patient assessment triangle
pED: Paediatric emergency department
PedCTAS: Pediatric Canadian triage and acuity scale
RETTS-p: Rapid emergency triage and treatment system for paediatrics
VS: Vital signs
